# Antibacterial microneedle patch releases oxygen to enhance diabetic wound healing

**DOI:** 10.1016/j.mtbio.2024.100945

**Published:** 2024-01-04

**Authors:** Mengli Sun, Xiqiang Zhong, Minghai Dai, Xujun Feng, Chengxuan Tang, Lingling Cao, Liangle Liu

**Affiliations:** a The Third Affiliated Hospital of Wenzhou Medical University, Wenzhou, 325200, China; b Jiujiang City Key Laboratory of Cell Therapy, The First Hospital of Jiujiang City, Jiujiang, 332000, China

**Keywords:** Silk fibroin methacryloyl, Microneedle, Diabetic wound healing, Calcium peroxide, Oxygen generation

## Abstract

Cell growth and metabolism require an adequate supply of oxygen. However, obtaining sufficient oxygen from the blood circulating around diabetic wounds is challenging. Nevertheless, achieving a continuous and stable oxygen supply is required for these wounds to heal. Hence, in this study, we report a novel antibacterial oxygen-producing silk fibroin methacryloyl hydrogel microneedle (MN) patch comprising tips encapsulated with calcium peroxide and catalase and a base coated with antibacterial Ag nanoparticles (AgNPs). The tip of the MN patch continuously releases oxygen and inhibits the production of reactive oxygen species. This accelerates diabetic wound healing by promoting cellular accretion and migration, macrophage M2 polarization, and angiogenesis. The AgNPs at the base of the MN patch effectively combat microbial infection, further facilitating wound repair. These findings suggest that using this multifunctional oxygen-producing MN patch may be a promising strategy for diabetic wound healing in clinical settings.

## Graphical abstract

**Figure:**
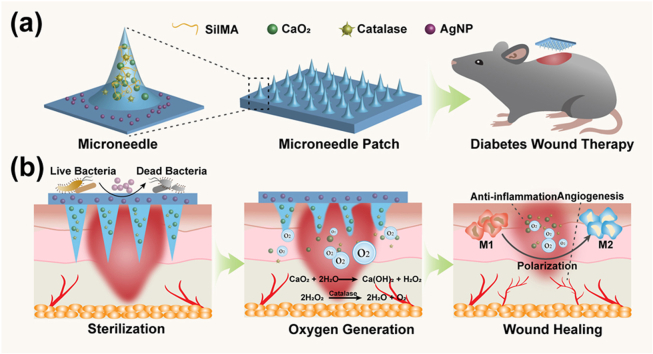

## 1 Introduction

Diabetic foot ulcers are the primary cause of hospitalization or amputation in patients with diabetes [1]. Delayed wound healing in these patients has serious implications, including persistent pain, ulceration, disability, and even life-threatening conditions [2]. The chronic high glucose state leads to complex pathological conditions, such as impaired blood microcirculation, immune deficiency, and excessive inflammation in diabetic wounds [3]. Impaired blood microcirculation impedes oxygen and nutrient supply to the wound, inhibiting re-epithelialization and extracellular matrix synthesis [4,5]. Moreover, the inflammatory response in the wound produces high oxygen consumption, further exacerbating hypoxia and creating a vicious cycle [6]. Moreover, over 55 % of diabetic foot ulcers are complicated by varying degrees of foot infections, resulting in increased severity and poor prognosis [7,8]. Therefore, relieving hypoxia and avoiding wound infection are necessary to promote diabetic wound healing.

Current therapies for improving oxygen supply to chronic diabetic wounds include hyperbaric chamber therapy, strategies to accelerate the formation of capillary networks (delivery of pro-angiogenic factors or overexpression of factors by stem cells), and use of hemoglobin-mimicking substances to transport oxygen [[9], [10], [11]]. However, these approaches have inherent disadvantages, including insufficient oxygen penetration and the relatively rapid release of oxygen [12,13]. It is, therefore, imperative to identify new therapeutic strategies to substantially alleviate the hypoxic microenvironment in diabetic wounds.

Hydrogel materials loaded with calcium peroxide (CaO_2_) can provide prolonged O_2_ release, thereby reducing tissue hypoxia and promoting tissue repair [14]. CaO_2_ reacts with water in the tissue to form H_2_O_2_, which is further converted to water and O_2_; H_2_O_2_ is a reactive oxygen species (ROS) that can cause severe tissue damage. Therefore, using catalase to reduce the risk of ROS generation is essential for preparing oxygen-releasing biomaterials [15,16]. Silk fibroin methacryloyl (SilMA) hydrogels offer advantages such as high biocompatibility, low cytotoxicity, and stable mechanical properties. Hence, they are widely used in biological tissue engineering involving bone, cartilage, and skin [17]. Moreover, microneedle (MN) patch systems are widely used for chronic wound healing owing to their rapid mode of action, low invasiveness, good patient compliance, and high penetration [18]. Hence, the use of CaO_2_-loaded SilMA hydrogel MNs as oxygen carriers represents a unique strategy for wound healing.

Infections are a leading cause of delayed healing in diabetic wounds. Persistent hyperglycemia provides favorable conditions for bacterial growth and invasion; these processes stimulate wound inflammation, leading to chronic refractory wounds. Hence, preventing bacterial infection is crucial in diabetic wound healing [19]. Moreover, Ag nanoparticles (AgNPs) have demonstrated excellent antimicrobial ability and biocompatibility [20] and, thus, can be incorporated in multifunctional oxygen-producing MN patches to confer antimicrobial properties.

**Fig. 1:**
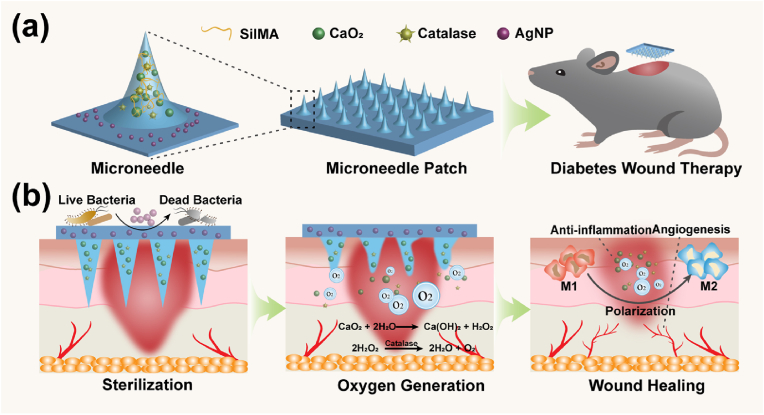
Antibacterial oxygen-generating microneedle (MN) patch for diabetic wound healing. (a) Schematic illustration of the MN patch. (b) Mechanism of the MN patch in wound healing.

Herein, we present a novel strategy to fabricate a multifunctional oxygen-producing SilMA hydrogel MN patch, with CaO_2_ and catalase loaded into the tip of each microneedle and the base of the patch coated with AgNPs (Fig. 1). After implantation into the diabetic wound, the CaO_2_ is gradually released from the microneedle tips, reacting with water from the interstitial tissue to produce calcium hydroxide [Ca(OH)_2_] and H_2_O_2_, which is broken down by catalase to form O_2_ and H_2_O. The slowly released oxygen improves the hypoxic state of the diabetic wound tissues, restoring disrupted cellular and immune system functions and remodeling blood vessels. Simultaneously, the AgNPs form an antimicrobial surface to prevent infection and, thus, promote wound healing. In diabetic wounds, the patch system stably adheres to the wound site and acts as an antimicrobial barrier, triggers angiogenesis, and reduces the inflammatory response, thereby accelerating healing. Collectively, our results demonstrate the potential of the MN patch for treating clinical diabetic wounds and other related diseases.

## 2 Results and discussion

### 2.1 MN@CaO_2_–AgNP patch design

In designing the oxygen-generating microneedle patch for diabetic wound treatment, CaO_2_ was selected as the substrate for oxygen production as its persistent and effective oxygen production has been demonstrated previously [[21], [22], [23]]. To avoid further tissue damage by the intermediate product, H_2_O_2_, 1 mg/mL catalase was incorporated as a scavenger to break down H_2_O_2_ into H_2_O and O_2_. SilMA was synthesized with primary silk fibroin (SF) amines chemically modified by glycidyl methacrylate (GMA). Fourier-transform infrared spectroscopy (FT-IR) identified spectra related to GMA, such as CHOH, CH_2_, and RRʹC=CH_2_ in SilMA, confirming the GMA modification of SF (Fig. S1).

The rheological properties of 5–20 % SilMA hydrogels were investigated. Under 0–1% strain conditions, the storage modulus (G′) was consistently higher than the loss modulus (G″) Figs. S2a and b(Fig. S2e and f). Similarly, at angular frequencies ranging from 1 to 65 rad/s and with a constant oscillation amplitude, the storage modulus (G′) remained higher than the loss modulus (G″) for all hydrogel concentrationsFigs. S2c and d(Fig. S2e and f). Within 30 s of UV curing, the storage module (G′) of 10–20 % SilMA hydrogels exceeded the loss module (G″). However, for the storage modulus (G′) of 5 % SilMA hydrogel to exceed the loss modulus (G″), UV curing for 1 min was required Figs. S2e and f(Fig. S2e and f). These findings suggest that the network structure of all SilMA hydrogels was stable (G' > G″) and that they possessed favorable elastic characteristics. SilMA (15 %) was selected as the base material for the microneedles owing to its beneficial physical properties, biocompatibility, and slow drug-releasing ability [6]. A SilMA solution loaded with CaO_2_ and catalase was prepared and used to encapsulate the microneedle tips. AgNPs at 4 mg/mL possess good antimicrobial properties and biosafety; thus, this AgNP concentration was used as the base substrate layer of the patch [24]. In this process, MN@CaO_2_–AgNP patches were prepared using polydimethylsiloxane (PDMS) negative molds.

**Fig. 2:**
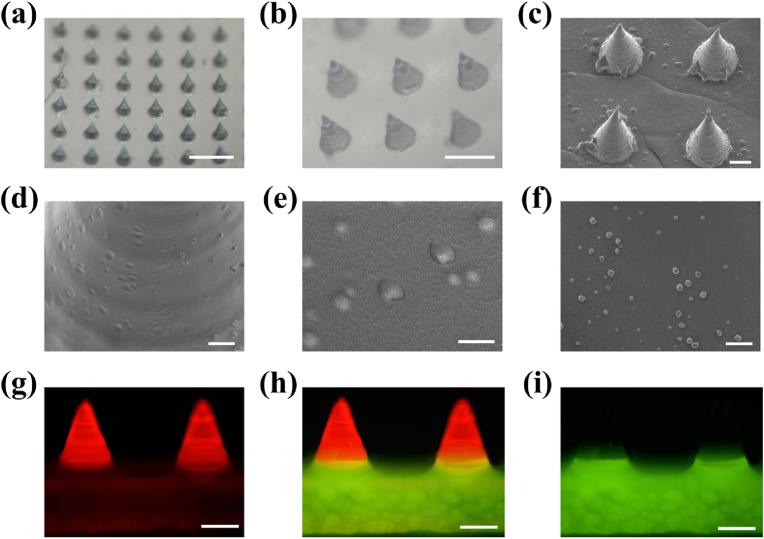
Characterization of MN@CaO_2_–AgNP patches. (a, b) MN patches examined under optical microscopy with intact and magnified images. SEM images of (c) the MN array, (d) magnified MN tips, (e) localized MN tips, (f) and floor of the MN back. (g, h, and i) Fluorescence images of the MN patch prepared using red fluorescent microsphere-loaded MN needle tips and a green fluorescent microsphere-loaded MN base. (a) Scale bars are 1 mm, (b) 500 μm, (c) 100 μm, (d) 10 μm, (e, f) 1 μm, and (g, h, and i) 200 μm. (For interpretation of the references to colour in this figure legend, the reader is referred to the Web version of this article.)

Fig. 2a and b shows the MN patches, in which all MNs were morphologically and geometrically sound. The 20 × 20 array MN had a tip height of 600 μm and tip-to-tip spacing of 750 μm, with a total area of 225 mm^2^. The MN tip morphology was observed by scanning electron microscopy (SEM); the tip shape was intact with a sharp point and rough surface (Fig. 2c and S3a). The high-magnification SEM image revealed a slightly uneven surface of the MN tips (Fig. 2d and e), which may be owing to different concentrations of CaO_2_ and H_2_O, leading to morphological changes in the tip surface. The SEM cross-sectional image revealed a solid MN tip structure (Fig. S3b); hence, no cavitations were formed within the MN tips during O_2_ generation. Moreover, the high-magnification SEM image of the MN basal layer showed numerous uniformly distributed particles (Fig. 2f). To confirm the successful two-step preparation method, the microsphere-loaded needle tip was stained with red fluorescence and the base was stained with green fluorescence and assessed via fluorescence microscopy (Fig. 2g–i).

Compositional analysis was performed to assess the loading of CaO_2_ and AgNPs in the MNs using energy-dispersive X-ray spectroscopy (EDX). The MN tips had pronounced Ca, O, and N element signals but not for Ag (Fig. S4a). The strong Ag elemental signal was detected in the MN base (Fig. S4b). The MN@CaO_2_–AgNP patch was immersed in phosphate-buffered saline (PBS) at 37 °C, and the solution was collected on days 0.5, 1, 3, 5, and 7 and subjected to the catalase activity assay to assess the capacity of catalase to scavenge H_2_O_2_. The MN@CaO_2_–AgNP patch exhibited good catalase activity that lasted for up to 7 days, peaking on day 3. Hence, the MN@CaO_2_–AgNP patch exhibits good sustainable release properties for catalase (Fig. S5a).

### 2.2 Oxygen release by MN@CaO_2_ patches

**Fig. 3:**
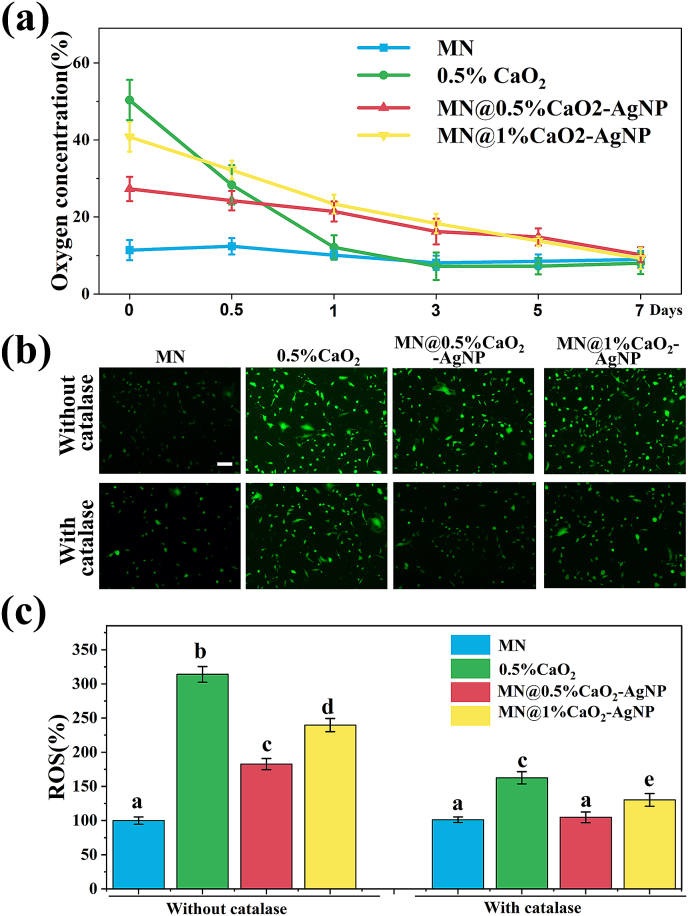
(a) Oxygen release kinetics of MN, 0.5%CaO_2_ solution, MN@0.5%CaO_2_-AgNP, or MN@1%CaO_2_–AgNP under hypoxic conditions. (b, c) ROS imaging and analysis of NIH-3T3 cells were performed using DCFH-DA after various treatments. Scale bars are 100 μm. Different letters denote statistically significant differences (p < 0.05).

A continuous slow release of oxygen was observed from MN@0.5%CaO_2_-AgNP and MN@1%CaO_2_–AgNP for up to 7 days. In contrast, the oxygen content of the 0.5 % CaO_2_ solution sharply decreased to normal levels within 24 h. The oxygen content of the MN@0.5%CaO_2_-AgNP hydrogel was slightly lower than that of the MN@1%CaO_2_–AgNP hydrogel after 24 h (Fig. 3a).

Given that excess intracellular ROS can be toxic and prolonged exposure can cause widespread intracellular damage [25], we examined the H_2_O_2_ content generated by CaO_2_ during oxygen production and its effect on NIH3T3 cells. To this end, we used MN patch immersion solution, 0.5 % CaO_2_ solution, MN@0.5%CaO_2_-AgNP immersion solution, and MN@1%CaO_2_–AgNP immersion solution, divided into groups with and without catalase. The H_2_O_2_ content assay revealed that the 0.5 % CaO_2_ solution produced the most H_2_O_2_, whereas the H_2_O_2_ content of the MN@0.5%CaO_2_-AgNP soaking solution was lower than that of the MN@1%CaO_2_–AgNP soaking solution with or without catalase. In the group treated with catalase, H_2_O_2_ production was effectively suppressed (Fig. S5b). Similarly, the 2,7-dichlorodihydrofluorescein diacetate (DCFH-DA) assay revealed that the 0.5 % CaO_2_ solution group had the highest ROS content in treated NIH3T3 cells. In contrast, the MN@0.5%CaO_2_-AgNP group had less ROS than the MN@1%CaO_2_–AgNP group with or without catalase. In the presence of catalase, the ROS content was much lower in all four groups (Fig. 3b and c).

Cellular activity assays were performed to verify the cytocompatibility of the hydrogels. In the medium containing catalase, the MN@0.5%CaO_2_-AgNP group caused no cellular damage. In contrast, the 0.5 % CaO_2_ solution group and MN@1%CaO_2_–AgNP group impaired the cellular activity of NIH-3T3 cells, with the 0.5 % CaO_2_ solution group showing the most significant damage (Fig. S5c). Hence, ROS production catalyzed by CaO_2_ reacting with H_2_O was the primary adverse effect elicited by the oxygen-producing hydrogel, leading to cell injury. However, the incorporated catalase effectively inhibited ROS production during oxygen production. In particular, MN@0.5%CaO_2_-AgNP loaded with catalase exhibited the best biocompatibility and was, therefore, selected for subsequent experiments.

### 2.3 Biocompatibility of MN@CaO_2_–AgNP patches

**Fig. 4:**
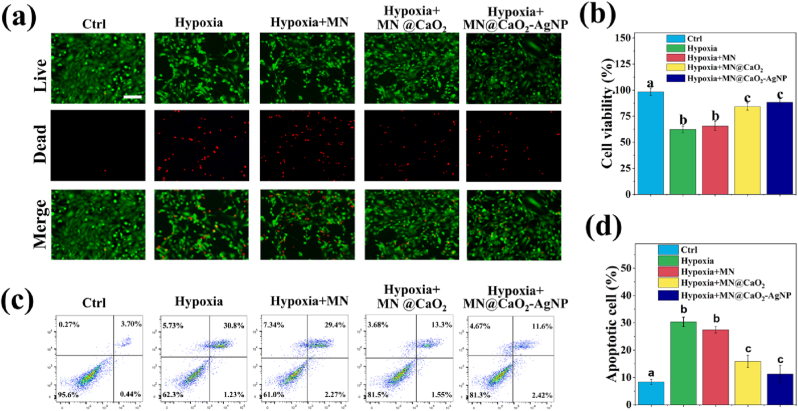
Biocompatibility of MN@CaO_2_–AgNP patches. (a, b) Stained NIH-3T3 cell fluorescence images indicating live (green) or dead (red) cells. (c, d) NIH-3T3 cell apoptosis following different treatments was analyzed using flow cytometry. Scale bars are 100 μm. Different letters denote statistically significant differences (p < 0.05). (For interpretation of the references to colour in this figure legend, the reader is referred to the Web version of this article.)

Hypoxia impairs cell survival and migration in diabetic wounds, leading to delayed wound healing [26]. Therefore, to assess the effect of oxygen released from MNs under hypoxic conditions on cell viability, we co-incubated NIH-3T3 cells with MNs in a hypoxic environment. Five experimental groups were included: control, hypoxic, hypoxic + MN, hypoxic + MN@CaO_2_, and hypoxic + MN@CaO_2_–AgNP. In the hypoxic microenvironment, the cellular activity of NIH-3T3 cells was significantly inhibited (Fig. 4a and b), and the apoptosis rate was significantly increased (Fig. 4c and d). The MN treatment group showed no significant damaging effects of hypoxia, indicating that SilMA is biocompatible, consistent with previous reports [27,28]. The MN@CaO_2_ and MN@CaO_2_–AgNP treatment groups improved hypoxia-induced effects, suggesting that the relevant tips effectively release dissolved oxygen, promote NIH-3T3 cell proliferation, and inhibit hypoxia-induced apoptosis. There was no significant difference between effects induced by MN@CaO_2_ and MN@CaO_2_–AgNP in NIH-3T3 cells, demonstrating the good biocompatibility of AgNPs coating the MN base.

### 2.4 Proangiogenic and antibacterial characteristics of MN@CaO_2_–AgNP patches

**Fig. 5:**
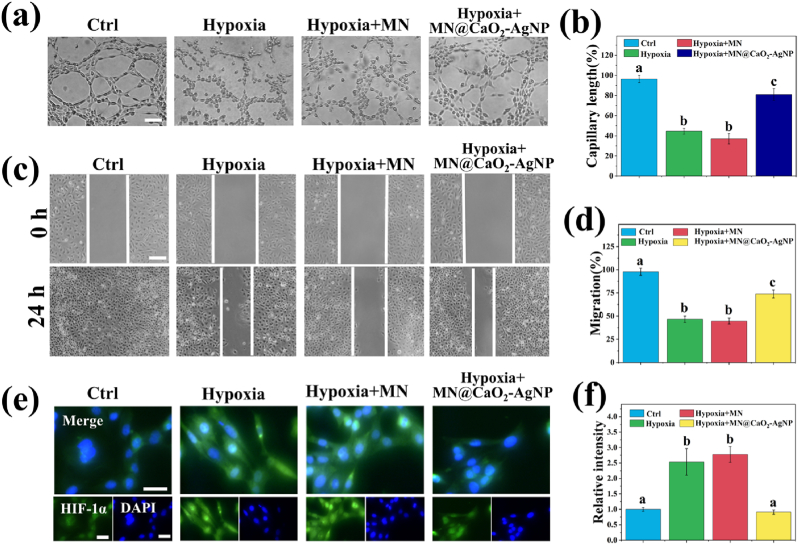
Ability of MN@CaO_2_–AgNP patches to migrate and form tubules was evaluated. (a) Formation of HUVEC tubes after 4 h of various treatments. (b) Analysis of tubule length results. (c) Cell migration images were obtained after scratching HUVEC cells and subjecting them to various treatments at 0 h and 24 h. (d) Quantitative analysis of cell migration by measuring gap sizes. (e) HIF-1α immunofluorescence and DAPI-stained merged images. (f) Analysis of HIF-1α fluorescence intensities. Scale bars are 100 μm. Different letters denote statistically significant differences (p < 0.05).

Angiogenesis plays an essential role in wound healing and affects the migratory capacity and tube-forming ability of endothelial cells [29]. Accordingly, wound healing and tube formation assays were performed to assess the pro-angiogenic ability of the patches. The inhibitory effect of hypoxia on tube formation was ameliorated by MN@CaO_2_–AgNP (Fig. 5a and b). Moreover, MN@CaO_2_–AgNP attenuated the inhibition of wound healing caused by hypoxia, whereas MN patches had no such effect (Fig. 5c and d).

Hypoxia-inducible factor-1⍺ (HIF-1⍺) is produced in response to hypoxia and helps promote angiogenesis and regulate the immune microenvironment [30]. Immunofluorescence results showed that hypoxia significantly induced HIF-1⍺ expression in human umbilical vein endothelial cells, whereas the MN@CaO_2_–AgNP patch reduced this effect (Fig. 5e and f). These results suggest that the MN@CaO_2_–AgNP patch effectively generates oxygen and alleviates angiogenesis suppression caused by hypoxia. The pro-angiogenic effect of MN@CaO_2_–AgNP was primarily due to its production of free oxygen rather than the effect HIF-1⍺.

The antibacterial capacity of the MNs was assessed via plaque coating and inhibition loop assays [31]. The inhibition ring was significantly larger after treatment with the MN@CaO_2_–AgNP soaking solution than in the control group and was the same size after treatment with the AgNP solution (Fig. S6a). Similarly, the results of the *Escherichia coli* and *Staphylococcus aureus* plate-coating assay confirmed that fewer colonies were observed after treatment with the MN@CaO_2_–AgNP soaking solution than in the control group and that the number was approximately the same with the AgNP solution (Figs. S6b and c). With MN@CaO_2_–AgNP, H_2_O_2_ produced at the MN tip may lead to the oxidation of AgNPs, resulting in the loss of antibacterial ability. However, the antibacterial ability of MN@CaO2–AgNP was similar to that of the AgNP solution, which is not affected by H_2_O_2_ at the MN tip. This may be because H_2_O_2_ is decomposed by catalase at the MN tips, which leads to little H_2_O_2_ entering the MN to oxidize AgNPs.

### 2.5 Wound healing and histology analyses in diabetic mice

**Fig. 6:**
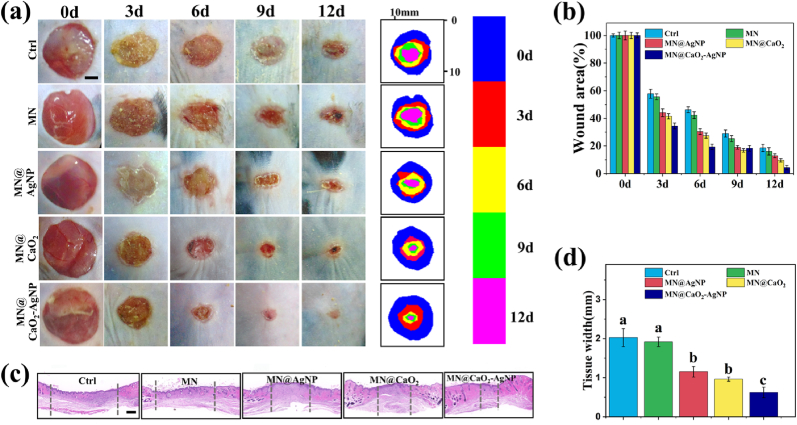
(a) Overall morphological appearance of the wound as it heals. Different colors represent different time intervals of the wound area map. (b) Percentage of the size of diabetic wounds over time. (c) Staining of wound healing tissue on day 12 with HE. (d) Width of granulation tissue observed using HE staining. (a) Scale bars are 2 mm and (b) 100 μm, respectively. Different letters denote statistically significant differences (p < 0.05). (For interpretation of the references to colour in this figure legend, the reader is referred to the Web version of this article.)

To evaluate the effect of the antibacterial oxygen-producing MN patch on tissue repair, a murine model of diabetes with infection was established. The wound surfaces were photographed at 0, 3, 6, 9, and 12 days. Wound healing was effectively promoted in the MN@CaO_2_, MN@AgNP, and MN@CaO_2_–AgNP groups from day 3 onwards. Among them, MN@CaO_2_–AgNP promoted healing significantly more than the MN@CaO_2_ and MN@AgNP groups (Fig. 6a and b). Hematoxylin and eosin (HE) staining of wound tissue sections on day 12 revealed that the MN@CaO_2_–AgNP group had the thinnest granulation tissue layer among the five groups, and the granulation tissue width in the MN@CaO_2_ and MN@AgNP groups was smaller than that in the control and MN groups (Fig. 6c and d). These results suggest that the MN patch containing AgNPs promotes wound healing in diabetic conditions by eliciting antibacterial effects. Moreover, the CaO_2_-containing MN tips promoted wound healing in the diabetic condition by slowly releasing oxygen. To further evaluate the antibacterial ability of MN@CaO_2_-AgNPs, we collected colonies from different treated mouse wounds on day 12 (Fig. S7). Significantly fewer colony-forming units (CFUs) were collected from the MN@AgNP and MN@CaO_2_–AgNP groups than from the control and MN groups. Interestingly, the MN@CaO_2_ group also had slightly fewer CFUs than the control and MN groups. This may have been due to CaO_2_ promoting skin healing, which may have inhibited bacterial growth. Hence, the MN components functioned collaboratively to accelerate wound healing in diabetic conditions.

### 2.6 Hemolysis and biocompatibility assays

A hemolysis test was performed to assess the effect of the antibacterial oxygen-producing MN patch on erythrocytes. Briefly, equal amounts of erythrocytes were added to PBS, MN patch, MN@CaO_2_ patch, and MN@CaO_2_–AgNP patch and incubated at 37 °C for 5 h, followed by photographic analysis (Fig. S8). The supernatant of the H_2_O group appeared deep red, whereas no significant color change was observed in the supernatant of the other groups.

The major internal organs (heart, liver, spleen, lungs, and kidneys) of diabetic mice were collected after MN treatment for HE staining to assess *in vivo* biocompatibility. No pathological changes, including necrosis, inflammation, or atrophy, were observed in any group (Fig. S9). Thus, we concluded that the MN@CaO_2_–AgNP patch exhibits good *in vivo* biocompatibility.

### 2.7 Pro-inflammatory factors in wound tissue

**Fig. 7:**
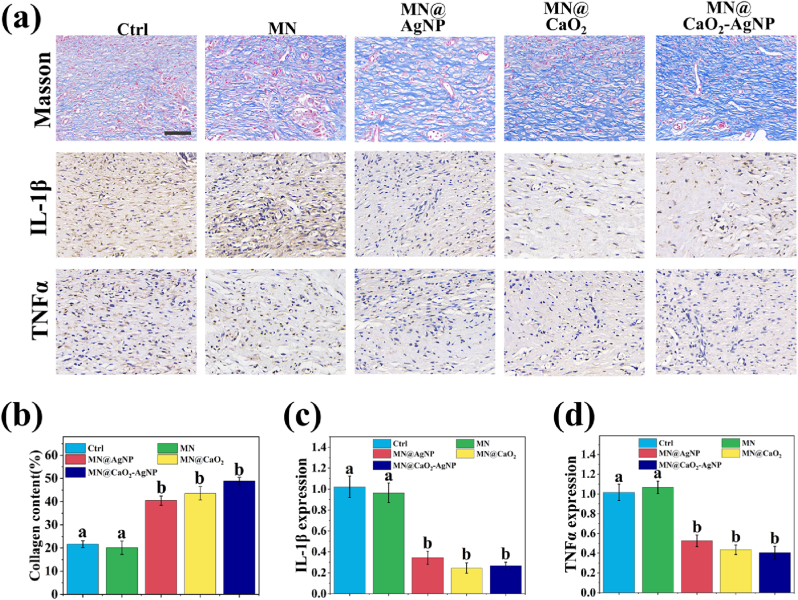
(a) Masson's trichrome staining and IL-1β and TNF-α visualization. (b, c, and d) Collagen content and expression of IL-1β and TNF-α. Scale bars are 200 μm. Different letters denote statistically significant differences (p < 0.05).

The chronic high expression of pro-inflammatory factors is a characteristic feature of chronic wounds [32], among which interleukin-1β (IL-1β) and tumor necrosis factor-α (TNF-α) are two major pro-inflammatory factors. Hence, the expression of IL-1β and TNF-α was measured on day 12 following diabetic wound treatment with MN patches to assess the inflammatory state. Immunohistochemistry showed the highest IL-1β and TNF-α levels in the control and MN groups, and lower expression in the MN@AgNP, MN@CaO_2_, and MN@CaO_2_–AgNP groups. Compared with that of MN@AgNP and MN@CaO_2_, the inhibitory effect of MN@CaO_2_–AgNP on cytokines was the most significant (Fig. 7a, c, and d).

Considering that collagen production contributes to wound healing and scar formation, collagen content was assessed using Masson's trichrome staining. The MN@CaO_2_–AgNP group exhibited the highest level of collagen among all groups (Fig. 7a and b), suggesting that MN@CaO_2_–AgNP patches promote wound healing under diabetic conditions.

### 2.8 Angiogenesis and macrophage polarization

**Fig. 8:**
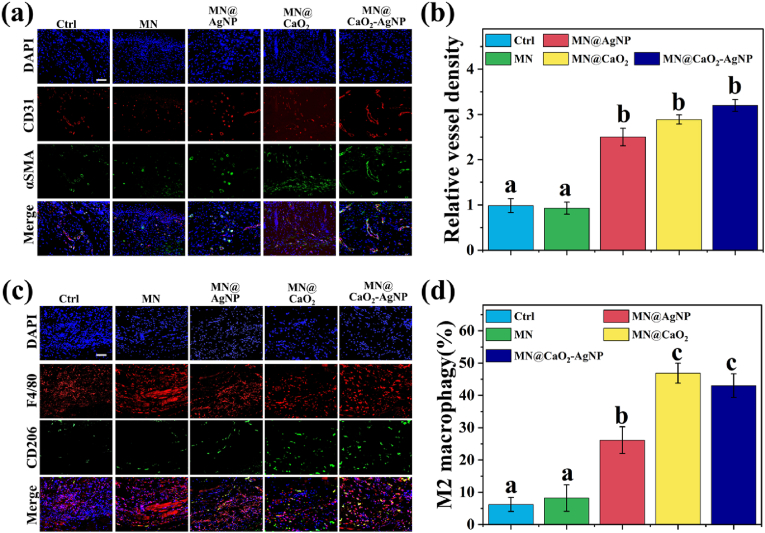
(a) Images of α-SMA (green) and CD31 (red) immunofluorescence staining. (b) Quantitative analysis of vascular density. (c) Images of CD206 (green) and F4/80 (red) immunofluorescence staining. (d) Analysis of the proportion of M2 macrophages. Scale bars are 200 μm. Different letters denote statistically significant differences (p < 0.05). (For interpretation of the references to colour in this figure legend, the reader is referred to the Web version of this article.)

Neovascularization improves nutrient and oxygen supply, contributing to wound healing. Hence, immunofluorescence staining using anti-CD31 and anti-α-SMA antibodies was performed to label blood vessels and assess neovascularization at the wound site [33,34]. Vascular density was significantly higher in the MN@AgNP, MN@CaO_2_, and MN@CaO_2_–AgNP groups than in the control and MN groups. The maximum vessel density was observed in the MN@CaO_2_–AgNP group (Fig. 8a and b). This was attributed to the oxygen-releasing effect of CaO_2_ and antibacterial effect of AgNPs. These results demonstrate that the MN@CaO_2_–AgNP patch promotes neovascularization.

Macrophage polarization to the M2 type is a benign event for accelerated wound tissue recovery [35]. Hence, we performed F4/80 and CD206 immunofluorescence staining to investigate the macrophage polarization status (total macrophages: F4/80^+^, M2 macrophages: F4/80^+^ and CD206^+^). The M2 macrophage ratio was increased in the MN@AgNP, MN@CaO_2_, and MN@CaO_2_–AgNP groups, with the highest proportion in the MN@CaO_2_–AgNP group (Fig. 8c and d). These results suggest that MN@CaO_2_-AgNPs promote macrophage M2 polarization in diabetes-associated wounds via oxygen release and infection control.

## 3 Materials and methods

### 3.1 Materials

SilMA was purchased from Engineering for Life Co., Ltd (Suzhou, China). CaO_2_ was provided by the School of Pharmaceutical Sciences of Central South University (Changsha, China). Catalase was obtained from PeproTech (Suzhou, China). AgNPs were purchased from Nanjing Nanoeast Biotech Co., Ltd. (Nanjing, China). HUVECs and NIH-3T3 cells were provided by Xiangya Central Laboratory (Changsha, China). Fetal bovine serum (FBS) and F12/Dulbecco's modified eagle medium (F12/DMEM) were procured from Gibco (Shanghai, China). The Annexin V FITC/PI apoptosis kit and Calcein/PI Assay Kit were purchased from Keygen Biotech Company (Nanjing, China). The Catalase Assay Kit was purchased from Beyotime Biotechnology (Shanghai, China). Matrigel was purchased from BD Biosciences (Shanghai, China). *S. aureus* and *E. coli* were provided by Beina Bio (Beijing, China). Eight-week-old male C57/BL mice were provided by Hunan Slaughter Jingda Laboratory Animal Technology Company (Changsha, China). All animal studies were approved by the Third Affiliated Hospital Ethics Committee of Wenzhou Medical University (Wenzhou, China).

### 3.2 FT-IR analysis

SF and SilMA samples were freeze-dried and ground into fine powder with potassium bromide (KBr). The spectra were recorded on the FT-IR spectrometer (Frontier, PerkinElmer, Rodgau, Germany) in the 4000 to 400 cm^−1^ range.

### 3.3 Rheological analyses

The rheological properties of the 5–20 % SilMA hydrogels were measured using a Discovery HR-2 rheometer (TA Instruments, New Castle, DE, USA). In brief, the hydrogel was placed on the test plate with a gap distance of 0.5 mm between the test plate and rotor (rotor diameter: 12 mm). The test temperature was maintained at 37 °C. The storage modulus (G′) and loss modulus (G″) of SilMA hydrogels at different concentrations were tested using the oscillation amplitude scanning method, starting with a fixed frequency of 1 Hz and strain range of 0–1%. Subsequently, the internal three-dimensional structure stability of the SilMA hydrogel was evaluated by assessing the modulus change in the angular frequency range of 0.1–100 rad/s, with a fixed strain of 1 %. For UV curing, SilMA hydrogels at different concentrations were irradiated with UV light for 5 min to measure the storage modulus (G′) and loss modulus (G″) at a frequency of 1 rad/s and target strain value of 0.1 %.

### 3.4 Fabrication of oxygen-generating MN@CaO_2_–AgNP patches

CaO_2_ was added to a DMSO solution containing 15 % SilMA, producing CaO_2_ at a concentration of 0.5 wt% or 1 wt%, which was then mixed by magnetic stirring at room temperature. The samples were freeze-dried at −80 °C to obtain SilMA@0.5% CaO_2_ or SilMA@1 % CaO_2_. A PBS solution (100 μL) containing 1 % (w/v) HMPP, catalase (1 mg/mL), and 15 % (w/v) SilMA@0.5% or 1 % CaO_2_ was poured onto the MN negative PDMS mold in vacuum to fill the tip of the conical microcavity with the hydrogel solution. After removing the excess solution, solidification was achieved by UV irradiation for 1 min. Subsequently, another 100 μL of PBS containing 1 % (w/v) HMPP, AgNPs (4 mg/mL), and 15 % (w/v) SilMA was added to cover the bottom layer of the MN patch. The hydrogel solidified after 2 min of UV irradiation. Finally, different MN patches were gently separated from the negative mold: MN patch without loading, MN@AgNP patch (MN base layer with loaded AgNPs [4 mg/mL], without MN tip), MN@CaO_2_ patch (MN@0.5% CaO_2_ and catalase [1 mg/mL] loaded in MN tip without MN base layer), MN@CaO_2_–AgNP patch (MN@0.5% CaO_2_ and catalase [1 mg/mL] loaded in MN tip with AgNPs [4 mg/mL] loaded in MN base layer), MN@0.5%CaO_2_-AgNP patch (MN@0.5% CaO_2_ loaded in MN tip with AgNPs [4 mg/mL] loaded in MN base layer), and MN@1%CaO_2_–AgNP patch (MN@1 % CaO_2_ loaded in MN tip with AgNPs [4 mg/mL] loaded in MN base layer).

### 3.5 Characterization of MN@CaO_2_–AgNP patches

The MN@CaO_2_–AgNP patches were scanned and photographed using SEM, and their microstructure and morphology were analyzed. Briefly, the MN@CaO_2_–AgNP patches were freeze-dried under a vacuum at −80 °C. Gold sputter coating was performed to improve the electrical conductivity of the freeze-dried MN patch samples before SEM analysis (Hitachi S–300 N, Japan). Patch compositional analysis was performed using EDX (Bruker, Quantax EDS Xflash 6/30, USA).

### 3.6 Measurement of CAT and oxygen concentration

To assess the CAT of the MN@CaO_2_–AgNP patch, it was placed in 0.5 mL of PBS at 37 °C. The soaking MN solution was obtained at 0.5, 1, 3, 5, and 7 days and catalase activity was assessed using a kit according to the manufacturer's protocol.

PBS was deoxygenated with nitrogen (N_2_) at room temperature. A CaO_2_ PBS solution (100 μL), MN, MN@0.5%CaO_2_-AgNP, or MN@1%CaO_2_–AgNP was added to 0.5 mL of deoxygenated PBS with or without catalase (1 mg/mL). Finally, a dissolved oxygen analyzer (JPB-607A, INESA Scientific Instruments, Shanghai, China) was used to measure the oxygen concentration of the sample at different time points in a nitrogen environment.

### 3.7 H_2_O_2_ measurements

A CaO_2_ PBS solution (100 μL), MN, MN@0.5%CaO_2_-AgNP, or MN@1%CaO_2_–AgNP was added to deoxygenated PBS (0.5 mL, with or without catalase [1 mg/mL]). All hydrogels were sealed and incubated at 37 °C for 24 h, protected from light. Subsequently, 50 μL of supernatant was collected from each sample, added to a 96-well plate, and processed using a hydrogen peroxide assay kit.

### 3.8 Cell culture and treatment

Normal NIH-3T3 cells and HUVECs were cultured in F12/DMEM containing 10 % FBS under a 5 % CO_2_/95 % air atmosphere. Hypoxic cells were cultured in serum-free and glucose-free DMEM under 5 % CO_2_/95 % N_2_ for 24 h. The extracting solution was prepared by immersing the MN, MN@CaO_2_, and MN@CaO_2_–AgNP patches in sterile PBS for 72 h. Cells that had grown to 70 % confluency were incubated with PBS, MN, MN@CaO_2_, and MN@CaO_2_–AgNP soaking solutions at 37 °C for 48 h with or without hypoxia treatment.

### 3.9 Cellular activity and apoptosis assays

For cellular activity assays, treated NIH-3T3 cells were stained with calcein-AM/PI for 10 min and then observed and photographed under a fluorescent inverted microscope.

For apoptosis assays, treated NIH-3T3 cells were stained using an Annexin-V FITC/PI kit and assayed via flow cytometry. Annexin V FITC^+^/PI^−^ indicated the proportion of cells in early apoptosis and Annexin V FITC^+^/PI^+^ indicated the proportion of cells in late apoptosis.

### 3.10 ROS detection

The DCFH-DA probe was used to detect ROS levels in NIH-3T3 cells after treatment. All samples were washed twice with PBS and stained with the DCFH-DA probe for 30 min at 37 °C. The cells were washed with PBS to remove excess DCFH-DA and observed under a fluorescent microscope.

### 3.11 Wound healing assays

HUVECs were plated in 6-well plates. When the cell density reached 100 %, the HUVEC monolayer was scraped using a 200-μL sterile pipette tip. The cell debris was washed with PBS and the scratched area was imaged 0 and 24 h after treatment.

### 3.12 Tubule formation analysis

Matrigel mixed in equal proportions with serum-free medium was inoculated into 96-well plates. The plates were incubated at 37 °C for 2 h. After various treatments, HUVECs (1 × 10^4^) were plated into 96-well plates containing Matrigel. Cells were incubated for 4 h at 37 °C, followed by photographic analysis and total tube length measurement using ImageJ.

### 3.13 Hemolysis assay

Ten milliliters of fresh blood was collected from each rat. Blood clotting was prevented by stirring with a glass rod. The erythrocytes were washed three times with PBS. After each wash, erythrocytes were collected by centrifugation. A 2 % erythrocyte suspension was prepared with PBS, and erythrocyte suspensions (5 mL) mixed with H_2_O, PBS, and MN patch, MN@CaO_2_ patch, or MN@CaO_2_–AgNP patch were incubated for 5 h at 37 °C.

### 3.14 Immunofluorescence analysis

Paraformaldehyde was used to fix NIH-3T3 cells after different treatments for 15 min. Cells were permeabilized and blocked for 30 min using a mixture of Triton X 100 (0.1 %) and sheep serum (10 %). Samples were incubated with the primary antibody (anti–HIF–1α; 1:300) for 12 h at 4 °C, washed with PBST, and incubated with the secondary antibody (1:200) for 2 h at room temperature, followed by DAPI staining. The samples were washed three times with PBST and images were captured using an inverted fluorescent microscope.

### 3.15 Animal experiments

Eight-week-old C57/BL6J mice were intraperitoneally injected with 60 mg/kg STZ for 5 consecutive days to establish a mouse model of type 1 diabetes mellitus (T1DM). The T1DM mouse model was declared successful after 1 week when the random blood glucose of the mice surpassed 16.7 mmol/L, accompanied with the symptoms of polydrinking, polyphagia, polyuria, and weight loss. Fifteen T1DM mice were randomly divided into five groups (*n* = 3/group) (control, MN, MN@CaO_2_, MN@AgNP, and MN@CaO_2_–AgNP groups). Following anesthesia, the dorsum of the mouse was manipulated to form a 1-cm wound, into which 0.1 mL of bacterial suspension was injected. The wounds were then treated with PBS or an MN, MN@CaO_2_, MN@AgNP, or MN@CaO_2_–AgNP patch. Mice were then placed in separate cages and provided *ad libitum* access to water and food. Wound size was calculated using ImageJ on days 0, 3, 6, 9, and 12 post-treatment. The wound closure rate (%) was calculated by dividing the original wound area by the residual wound area at certain intervals. On day 12 of treatment, all mice were humanely euthanized, and the regrown tissue was excised for histological experiments. Heart, liver, spleen, lung, and kidney tissues were collected and subjected to hematoxylin and eosin (HE) staining.

### 3.16 Antimicrobial activity

The disc diffusion method was used to determine antimicrobial activity, as previously described [36]. Briefly, 1 × 10^8^ CFU/mL of *E. coli* or *S. aureus* suspension was inoculated onto LB agar plates and spread evenly. Round filter paper (5 mm) was impregnated with a PBS, MN, AgNP, or MN@AgNP soaking solution for 5 min and then placed on the plate containing sterile medium. Thereafter, the dishes were inverted and incubated at 37 °C overnight. The size of each inhibition ring around the impregnated filter paper was observed.

Next, the plate coating method was used. Briefly, a 1 × 10^8^ CFU/mL bacterial suspension was mixed with PBS, MNs, AgNPs, or MN@CaO_2_-AgNPs and incubated at 37 °C for 24 h. The bacterial suspension was diluted 1000 times and inoculated onto the surface of a Petri dish containing solidified agarose and incubated at 37 °C for 24 h. The Petri dish was removed and photographed for counting. To assess wound infection in mice, wounds were swabbed, the swabs were washed in PBS, spread evenly on LB agar plates, and cultured for 18 h at 37 °C; finally, CFUs were counted manually.

### 3.17 Wound microenvironment assessment

The wound tissue was first subjected to histological experiments. To avoid errors caused by sectioning at different positions, tissue sections were cut along the longest axis of the wound. HE staining and Masson's trichrome staining were used to determine intra-tissue healing and collagen content, respectively. Immunohistochemical experiments using anti-IL-1β and anti-TNF-α antibodies were performed to assess inflammation. Immunofluorescence experiments were performed using anti-CD31 and anti-α-SMA antibodies to analyze neovascularization. Macrophages in the wound tissue were labeled for F4/80 and CD206: total macrophages stained F4/80^+^, M2 macrophages stained F4/80^+^ and CD206^+^.

### 3.18 Statistical analysis

Data are reported as mean ± standard error. The Student's *t*-test, analysis of variance (ANOVA), and Fisher's least significant difference tests were used for independent samples. A statistical significance level of *p* < 0.05 was set. All analyses were performed using SPSS Statistics (version 23.0; IBM, USA).

## 4 Conclusion

Here, we report a potential strategy for treating chronic diabetic wounds with a SilMA hydrogel patch containing CaO_2_ and catalase in the MN tip and antibacterial AgNPs at the base. This multifunctional, self-oxygenating carrier supplies sufficient oxygen directly to the deep skin layers to promote diabetic wound healing, thereby successfully addressing the limitation of restricted tissue gas permeability in currently used treatment modalities. Our therapeutic hydrogels exhibit good antibacterial properties, thereby effectively avoiding bacterial infections that may impede wound healing. In addition, the MNs can scavenge free radicals and slow wound inflammation. Notably, our MNs showed good therapeutic effects in treating skin wounds in a co-infected mouse model of T1DM. In conclusion, the designed hydrogel patch offers good biocompatibility, multifunctionality, and high stability, making it a promising therapeutic modality for treating diabetic wounds and for application in related biomedical fields.

## CRediT authorship contribution statement

**Mengli Sun:** Writing - original draft, Investigation, Conceptualization. **Xiqiang Zhong:** Visualization, Project administration. **Minghai Dai:** Writing - review & editing. **Xujun Feng:** Visualization, Software, Investigation. **Chengxuan Tang:** Investigation, Data curation. **Lingling Cao:** Supervision, Resources, Conceptualization. **Liangle Liu:** Validation, Methodology, Conceptualization.

## Declaration of competing interest

The authors declare that they have no known competing financial interests or personal relationships that could have appeared to influence the work reported in this paper.

## Data Availability

Data will be made available on request.
