# Supplementary material for: Antibacterial microneedle patch releases oxygen to enhance diabetic wound healing
Source: Mater Today Bio. 2024 Jan 4;24:100945. doi: 10.1016/j.mtbio.2024.100945 (PMC10789642; doi:10.1016/j.mtbio.2024.100945)
Supplement: Multimedia component 1 [file mmc1.docx]

**Supplementary material**

**[Antibacterial](javascript:;) Microneedle with Oxygen Generation for Diabetic Wound Healing**

Mengli Sun^1^, Xiqiang Zhong^1^, Minghai Dai^1^, Xujun Feng^2^, Chengxuan Tang^1^, Lingling Cao^2,*^, Liangle Liu^1,*^

^1^The Third Affiliated Hospital of Wenzhou Medical university, Wenzhou, 325200, China

^2^Jiujiang City Key Laboratory of Cell Therapy, The First Hospital of Jiujiang City, Jiujiang, 332000, China

*Corresponding author.

Email address: liuliangle@wmu.edu.cn (Liangle Liu)

1309316573@qq.com (Lingling Cao)


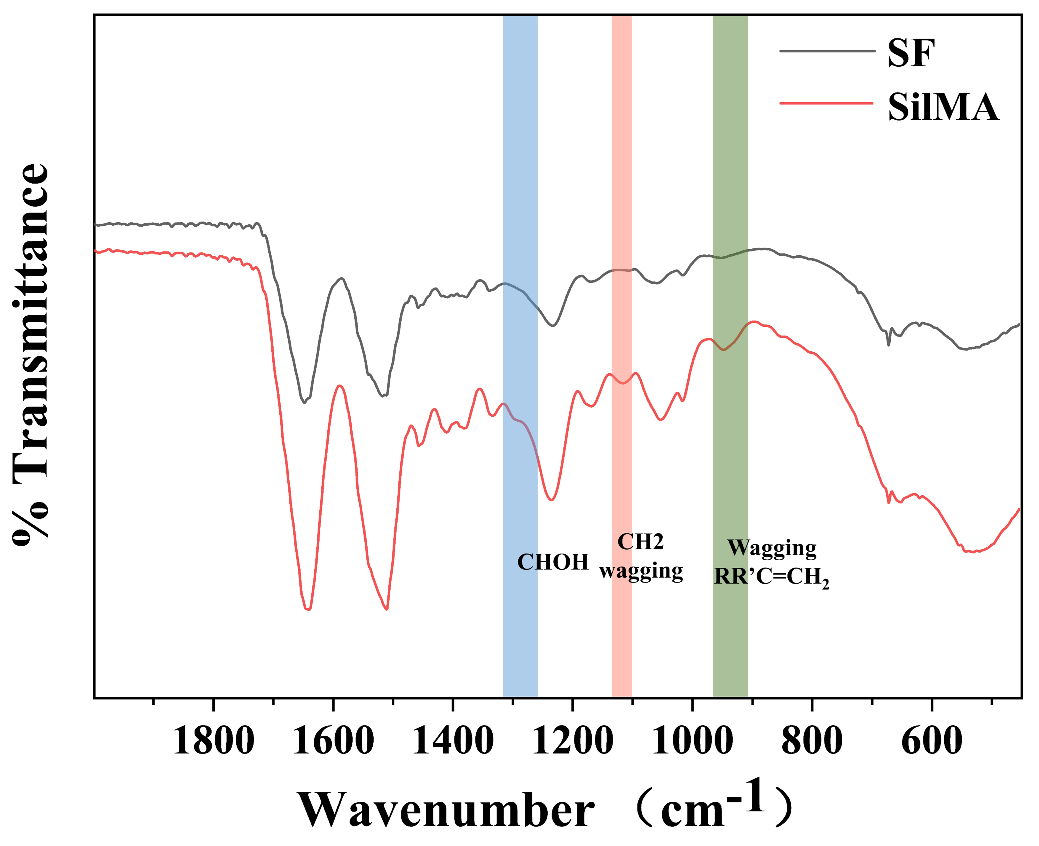


**Figure S1.** FT-IR spectra unsubstituted SF and SilMA.


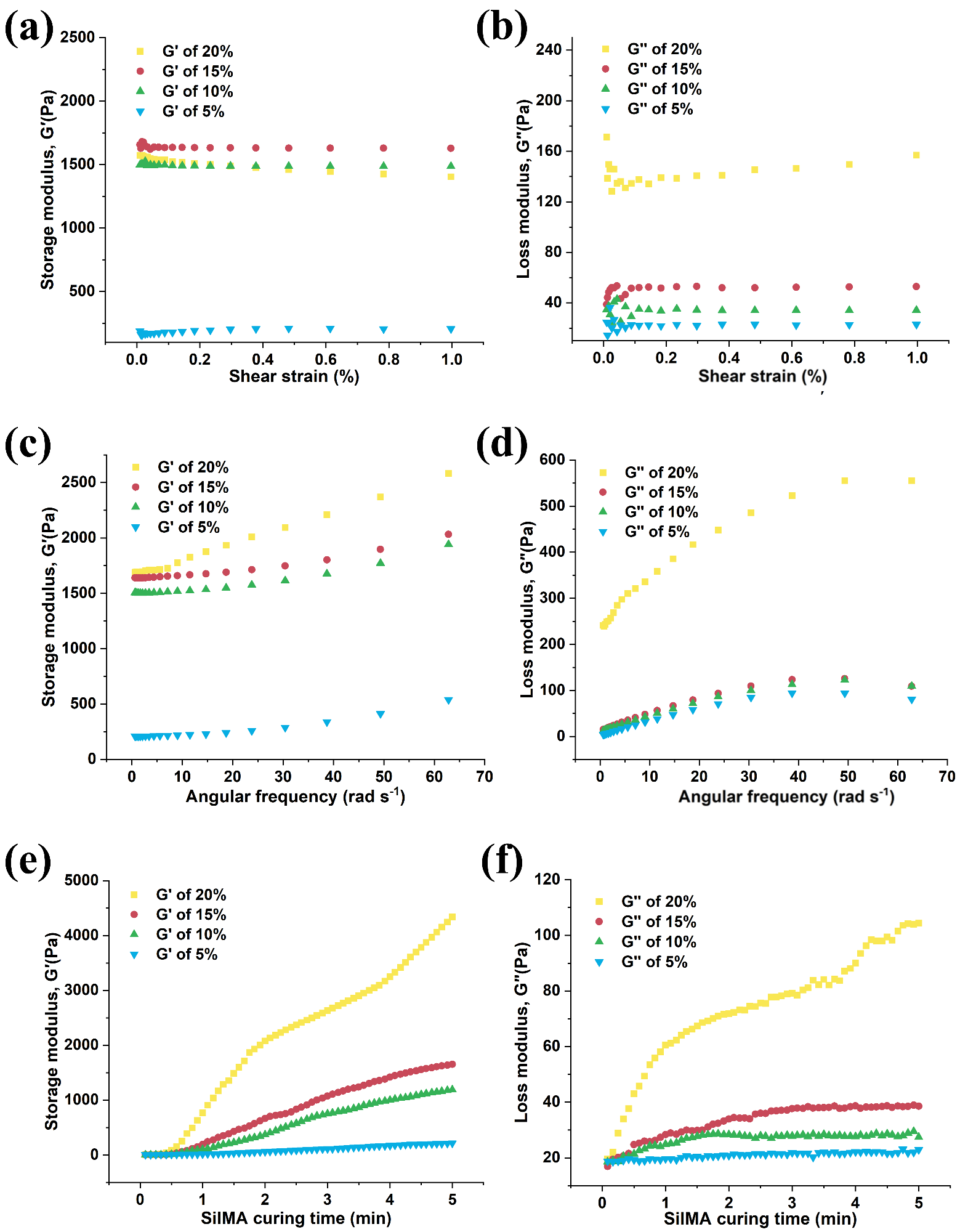


**Figure S2.** Rheological analysis for SilMA hydrogel. Strain dependency (a, b) and frequency dependency (c, d) of storage modulus (G′) and loss modulus (G″) for SilMA hydrogels at different SilMA contents varied from 5 to 20%. The effect of SilMA contents on the storage modulus (G′) and loss modulus (G″) during UV exposure for 5min (e, f).


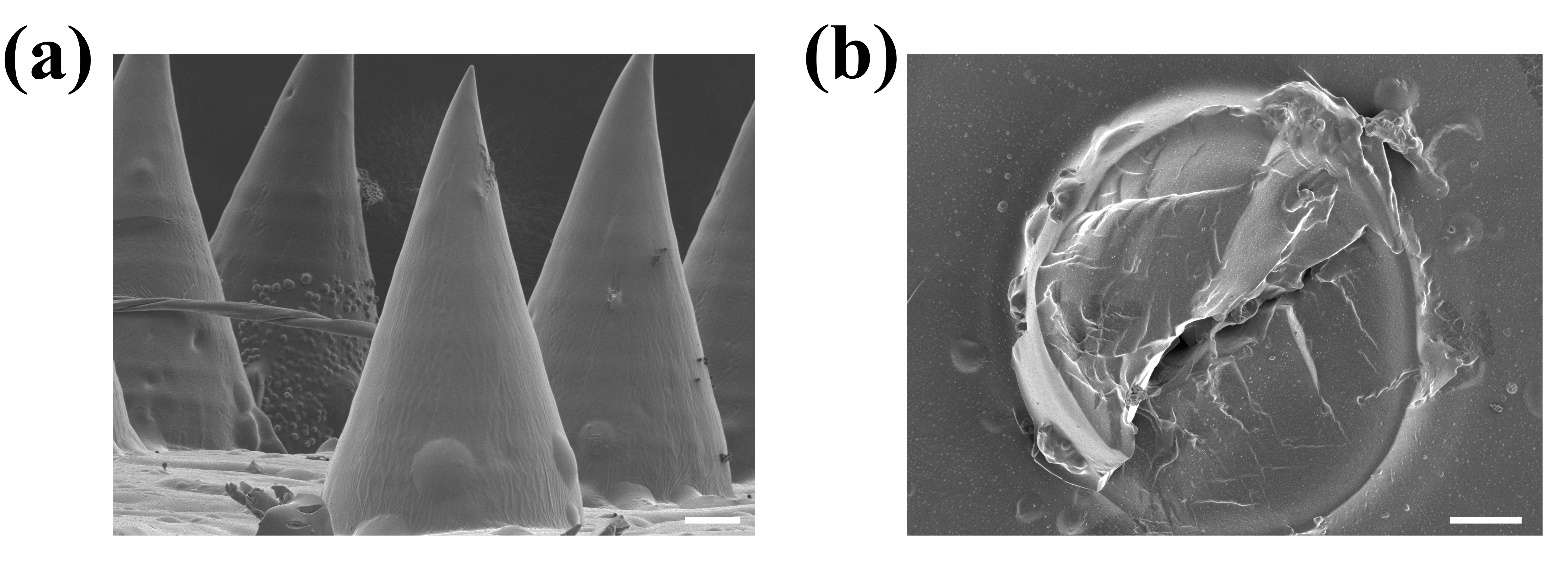


**Figure S3.** Scanning electron microscopy images of the MNs with (a) side views and (b) fracture surface sections. (a) Scale bars represent 50 μm and (b) 40 μm, respectively.


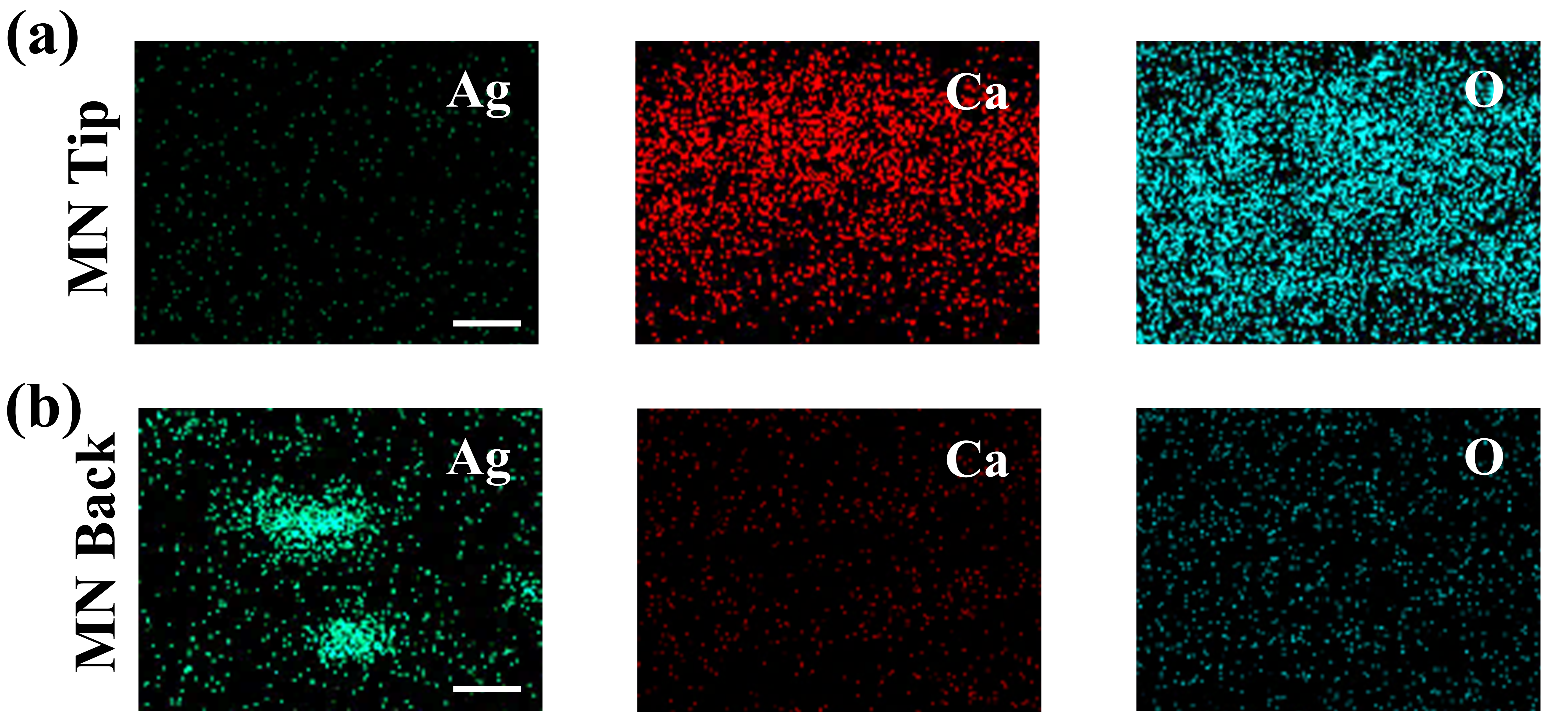


**FigureS4.** Element mapping images of Ag, Ca, and O in MN tip (a) and MN back (b). Scale bars respectively are 1 μm.


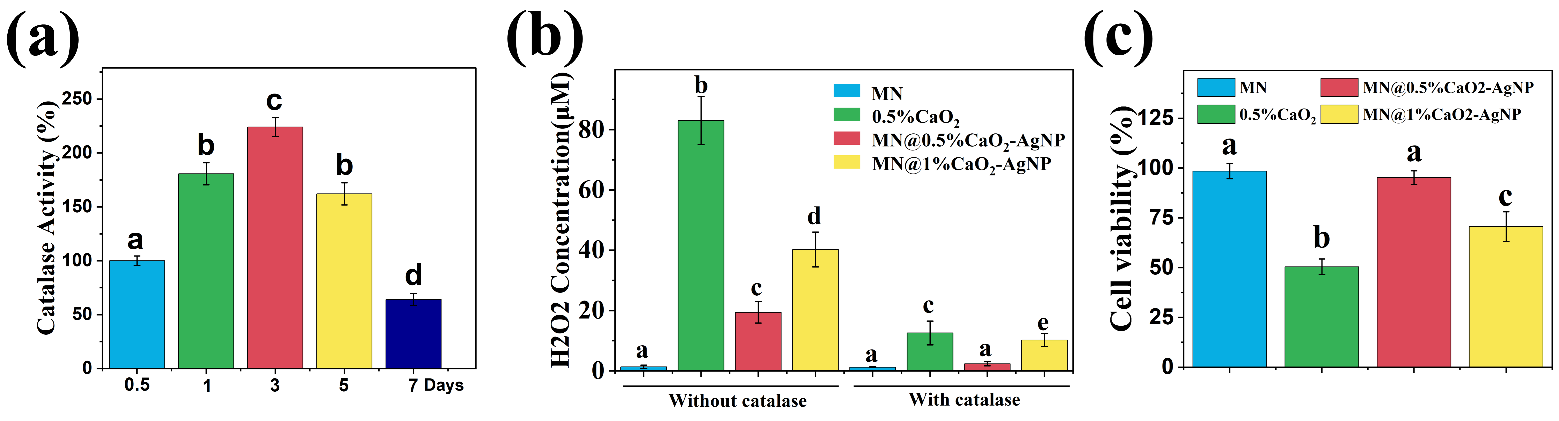


**Figure S5.** (a) Catalase activity of MN@CaO_2_-AgNP. (b) H_2_O_2_ release profile with and without catalase under different treatments. (c) Cell activity analysis using a live/dead staining assay. The groups with different alphabetic letters denote statistically significant differences (p < 0.05).


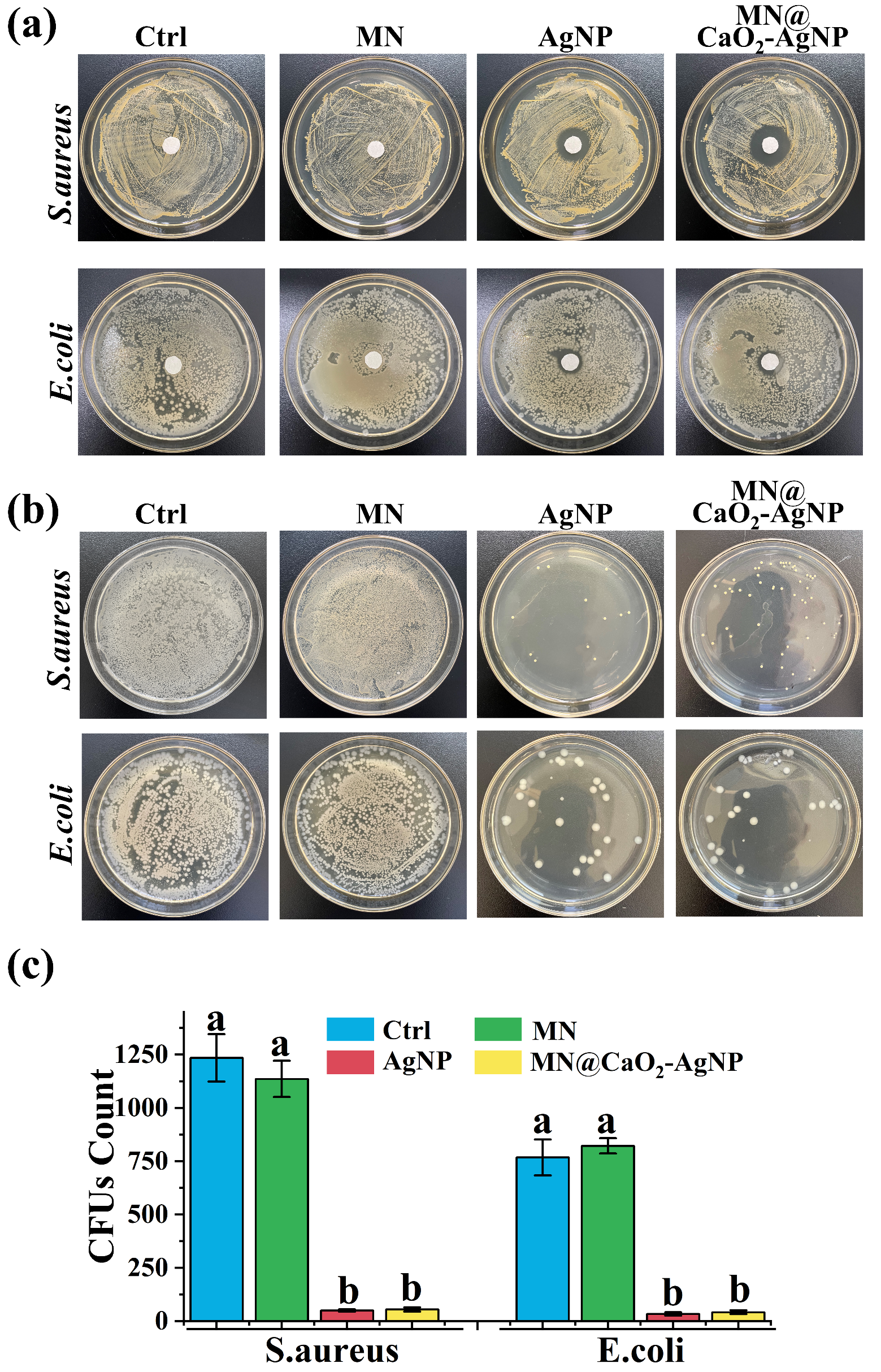


**Figure S6.** (a) The number of *E.coli* or *S. aureus* colonies on agar plates after exposure to PBS, MN, AgNP, and MN@CaO_2_-AgNP treatments. (b, c) Agar plates showing zones of inhibition around PBS, MN, AgNP, and MN@CaO_2_-AgNP. The groups with different alphabetic letters denote statistically significant differences (p < 0.05).


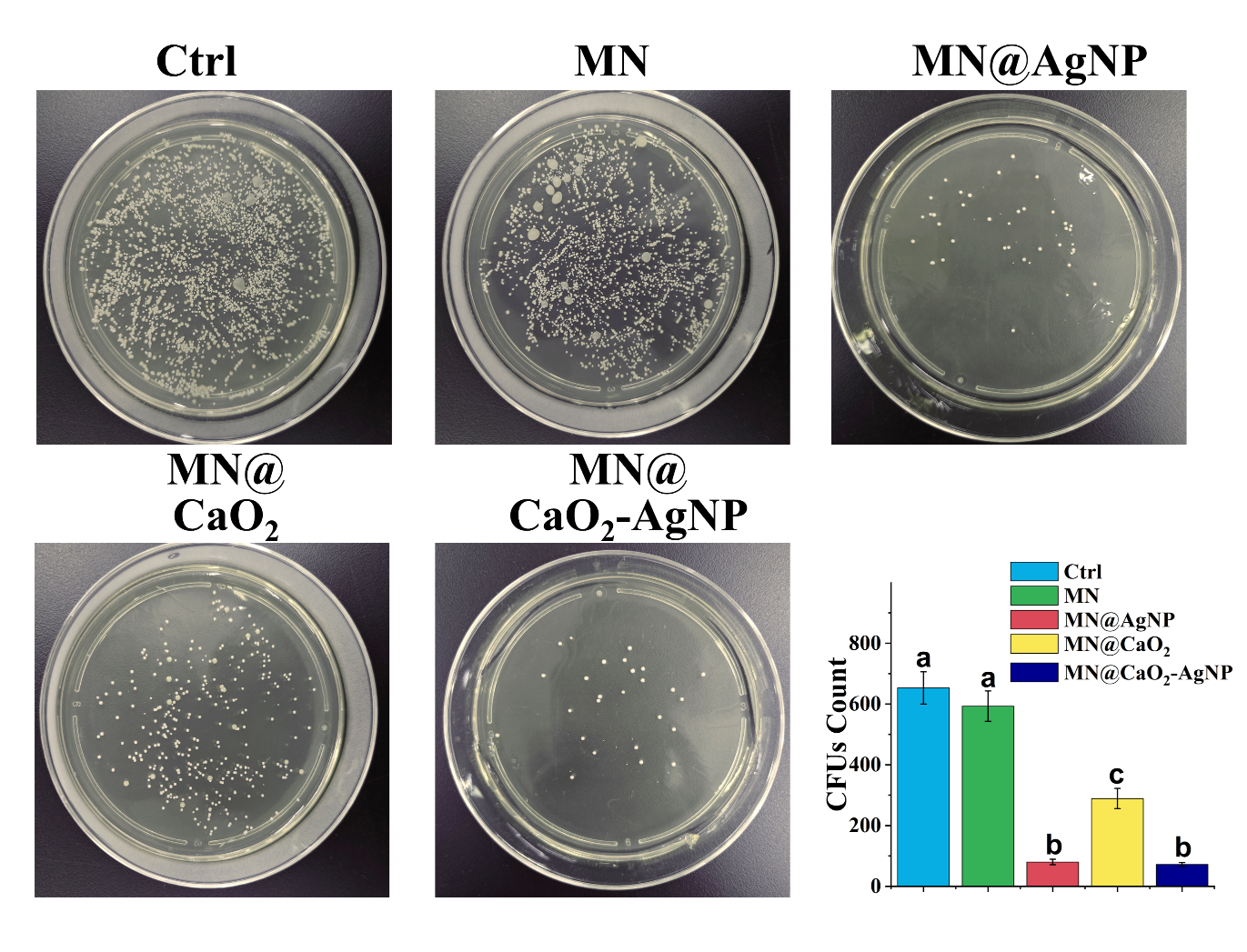


**Figure S7.** The colonies from different treated mouse wounds on 12 days. The groups with different alphabetic letters denote statistically significant differences (p < 0.05).


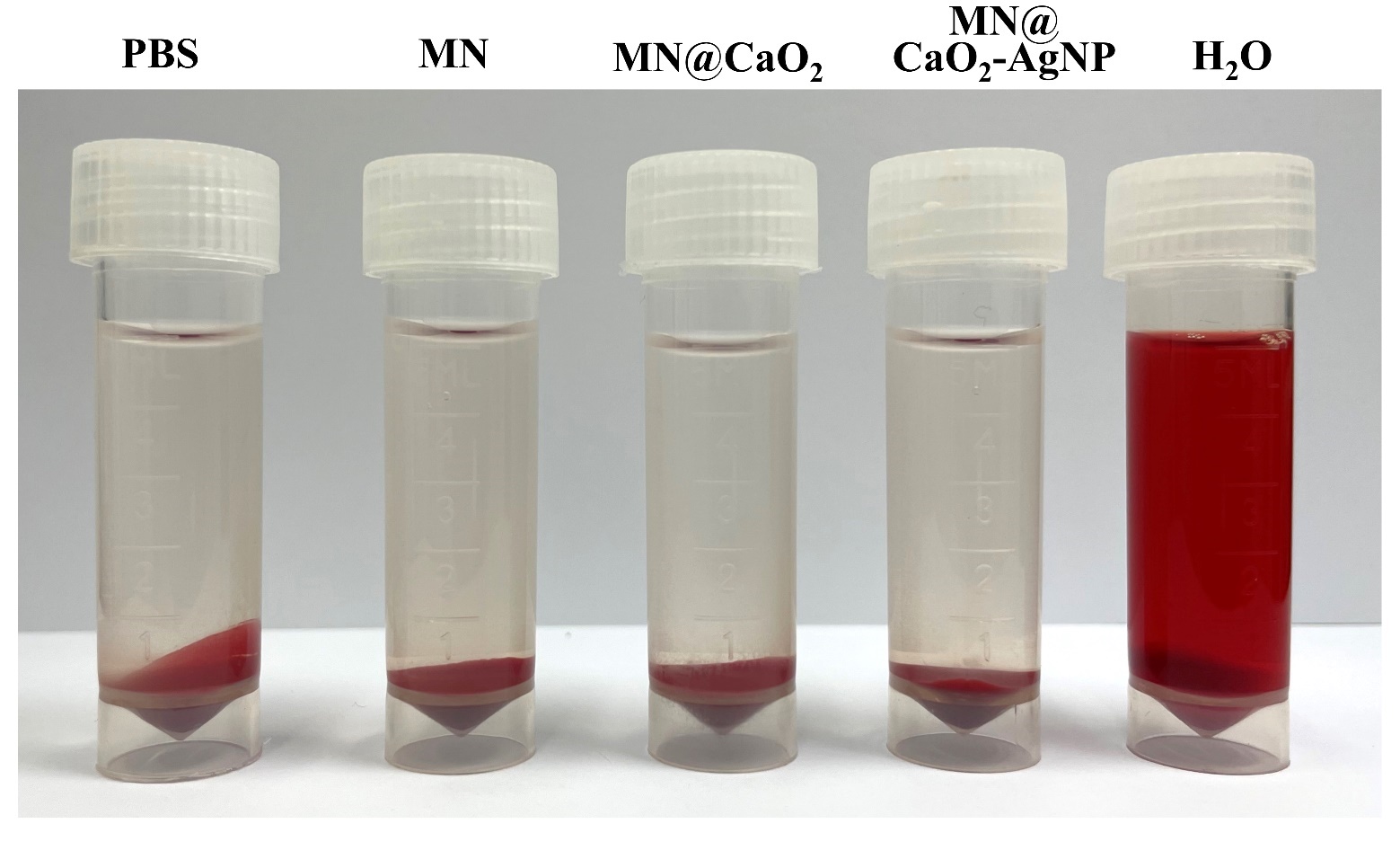


**Figure S8.** Hemolysis testing showing the appearance of each group of samples in erythrocyte suspension at 5 h.


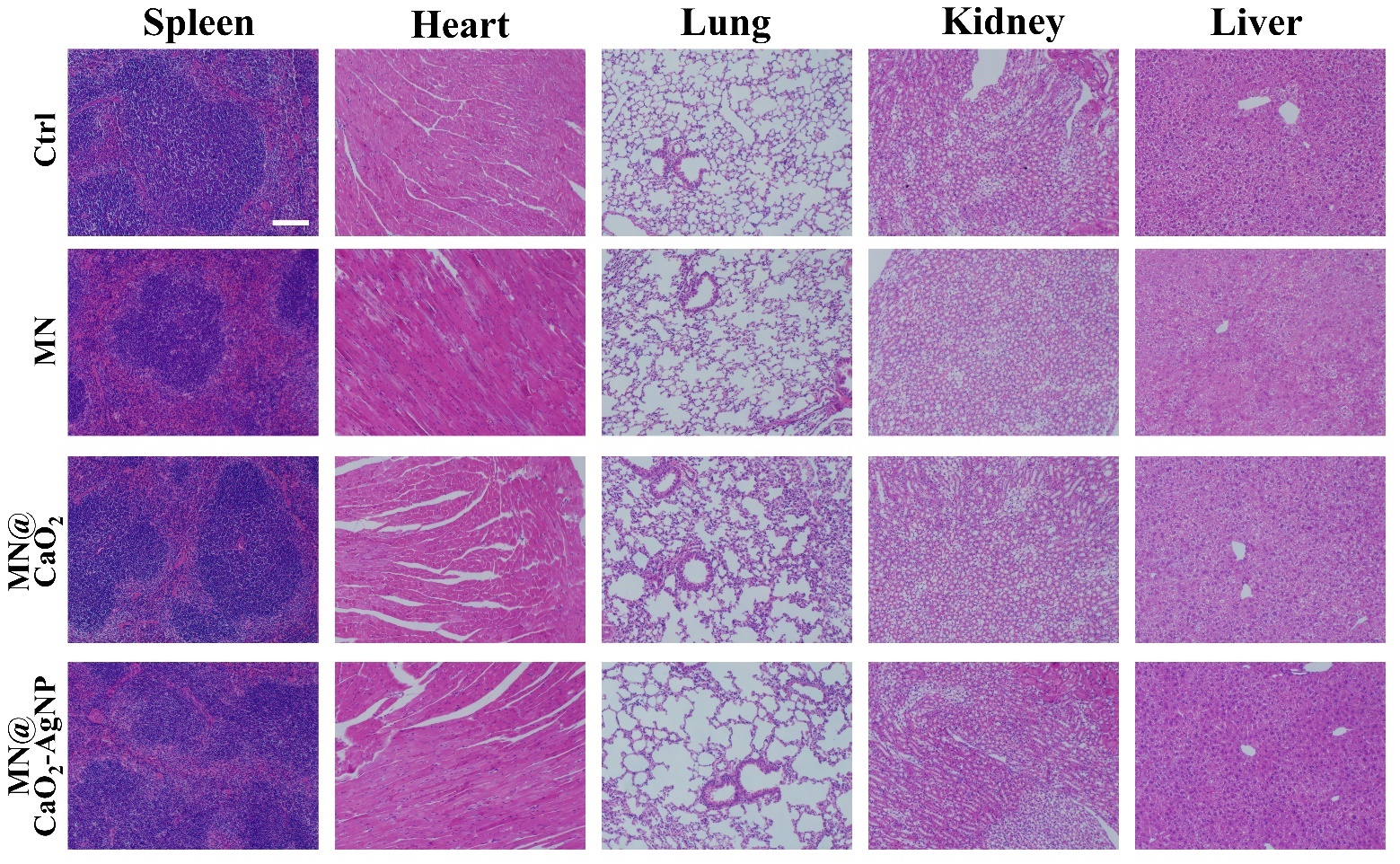


**Figure S9.** Hematoxylin and eosin (HE) staining of surrounding tissues of mice in different groups after treatment for nine days. Scale bars are 200 μm.
